# A case of pharyngeal injury in a patient with swallowed toothbrush: a case report

**DOI:** 10.1186/1756-0500-7-788

**Published:** 2014-11-06

**Authors:** Yeon-Hoo Kim, Sung-Il Cho, Nam-Yong Do, Jun-Hee Park

**Affiliations:** Department of Otolaryngology-Head and Neck surgery, Chosun University, 365 Pilmun-daero, Dong-gu, Gwanju, 501-717 South Korea

**Keywords:** Toothbrush, Foreign body, Hypopharynx perforation

## Abstract

**Background:**

Otolaryngologists encounter cases of various foreign bodies in the oral and pharyngeal regions. One commonly found foreign body is a fish bone, ingested in most cases by carelessness or an accident. These foreign materials are removed by endoscopy or through a simple procedure. However, hypopharyngeal damage is rarely caused by a foreign body in the pharynx following the swallowing of a toothbrush.

**Case presentation:**

A 44-year-old Asian male visited the emergency room with chief complaints of intraoral pain and dysphagia that had started on the same day. The patient had paranoid-type schizophrenia that began 10 years ago; he had been hospitalized and was being treated at another clinic, and was transferred to the emergency room by the medical staff after swallowing a toothbrush. We successfully removed a toothbrush located within the pharynx of a patient with a history of a psychologic disorder via surgery and conservative treatment.

**Conclusion:**

The case with this patient, and a rapid diagnosis as well as treatment is imperative. The presence and state of a foreign body must be determined through a careful physical examination and imaging, followed by the immediate removal of the foreign body, all while keeping in mind the possibility of accompanying damage to nearby tissues.

## Background

Foreign bodies are a common problem frequently faced by otolaryngologists. In cases of ingestion of foreign bodies such as fish bones, removal can be easily performed through simple manipulation. However, in some cases of foreign bodies swallowed by children or unusual foreign bodies swallowed by adults, removal via surgery under general anesthesia is necessary. Amongst a variety of foreign bodies, a toothbrush typically triggers gastrointestinal tract problems, but no resultant oropharynx or hypopharynx damage has been reported, as was the case with this patient [1]. We hereby report a case, with a literature review, of treatment via surgery for accidental toothbrush swallowing by a patient with a history of a psychological disorder, and the conservative treatment of oropharyngeal and hypopharyngeal damage caused by the toothbrush.

## Case presentation

**Figure 1:**
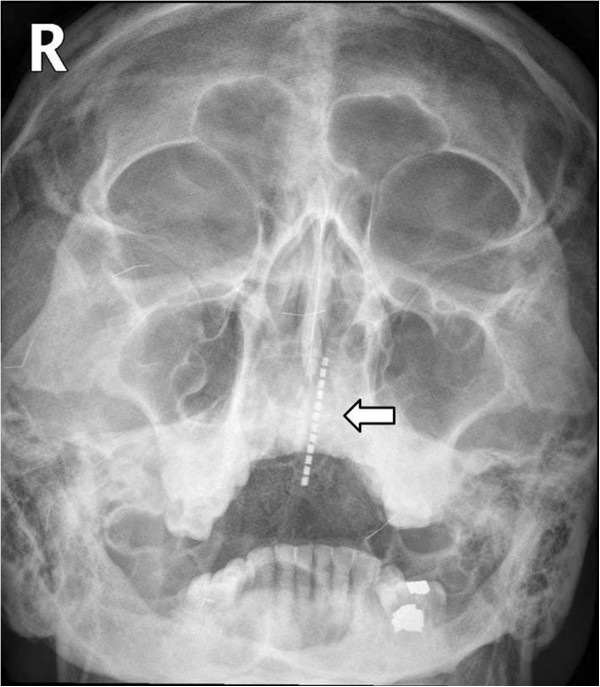
**(Arrow) A toothbrush head observed in the nasal cavity and oropharynx region.** (Water’s view).

**Figure 2:**
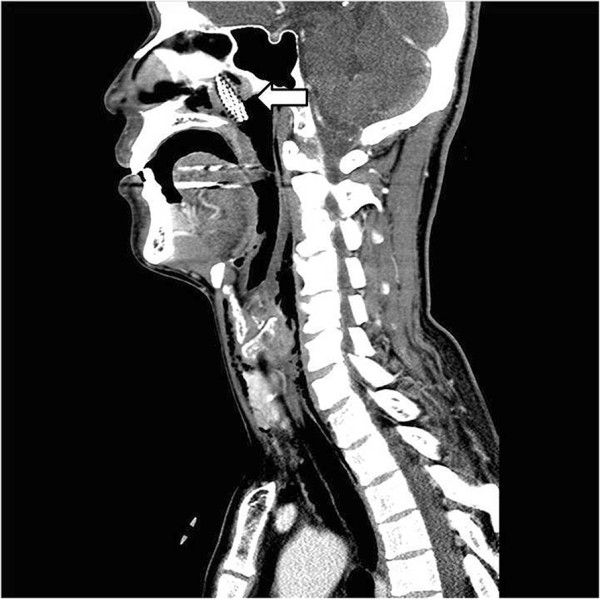
**Sagittal view of neck CT scan.** (Arrow) A neck CT shows the toothbrush spanning the nasopharynx and oropharynx, as well as subcutaneous emphysema in the anterior and lateral cervical regions.

**Figure 3:**
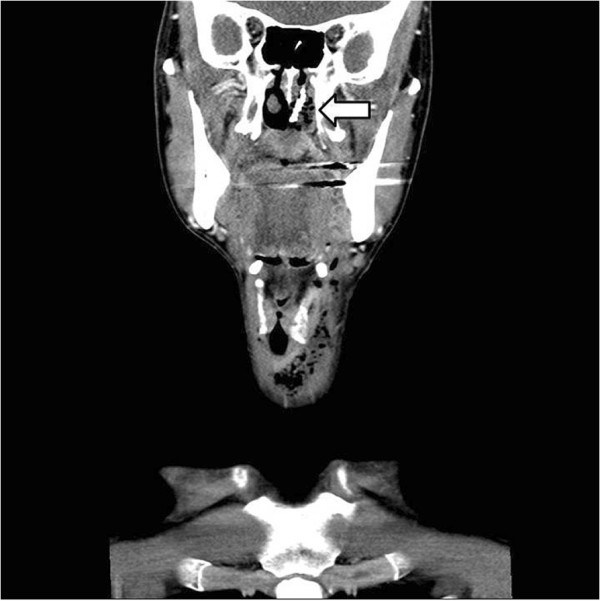
**Coronal view of same CT.** (Arrow) A neck CT shows the toothbrush spanning the nasopharynx and oropharynx, as well as subcutaneous emphysema in the anterior and lateral cervical regions.

A 44-year-old Asian male visited the emergency room with chief complaints of intraoral pain and dysphagia that had started on the same day. The patient had paranoid-type schizophrenia that began 10 years ago; he had been hospitalized and was being treated at another clinic, and was transferred to the emergency room by the medical staff after swallowing a toothbrush.At the time of admission, the patient’s mental status, including orientation, was alert, and other vital signs were stable as well. Physical examinations showed uvular lacerations and a foreign body that appeared to be a toothbrush in the posterior oropharyngeal wall. A plain radiograph of the paranasal sinuses confirmed the presence of a foreign body located in the nasal cavity and oropharynx (Figure 1). CT scan of the neck revealed a foreign body spanning from the left nasopharynx to the right hypopharynx, as well as a broad area of subcutaneous emphysema in the anterior and lateral cervical regions (Figures 2 and 3). As additional damage could occur if the foreign body removal process was delayed, an emergency surgery was attempted.

**Figure 4:**
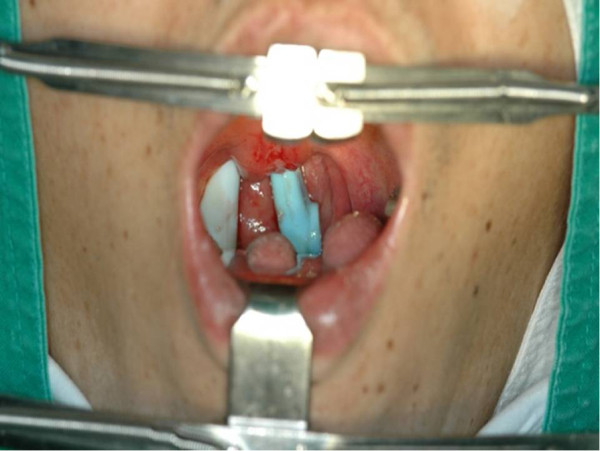
**At the oropharynx, part of the toothbrush handle is observed.**

**Figure 5:**
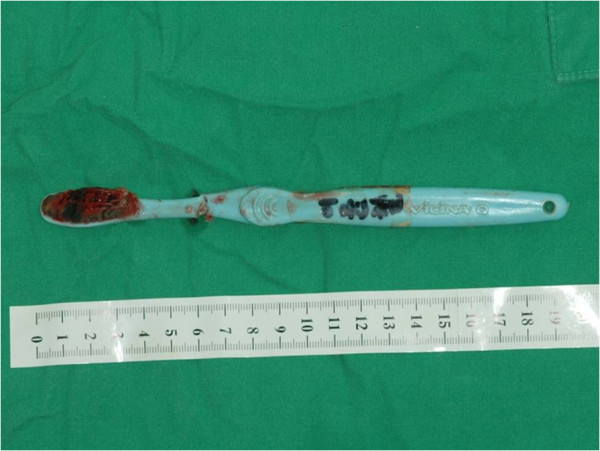
**The removed toothbrush after the surgery.** Total length is approximately 20 cm.

An intraoral approach employing nasotracheal tube insertion under general anesthesia was used. The exposed part of the toothbrush was cut with an electric surgical saw. Following the removal of the two separate parts, the uvular laceration was sutured and the surgery was completed (Figures 4 and 5).

**Figure 6:**
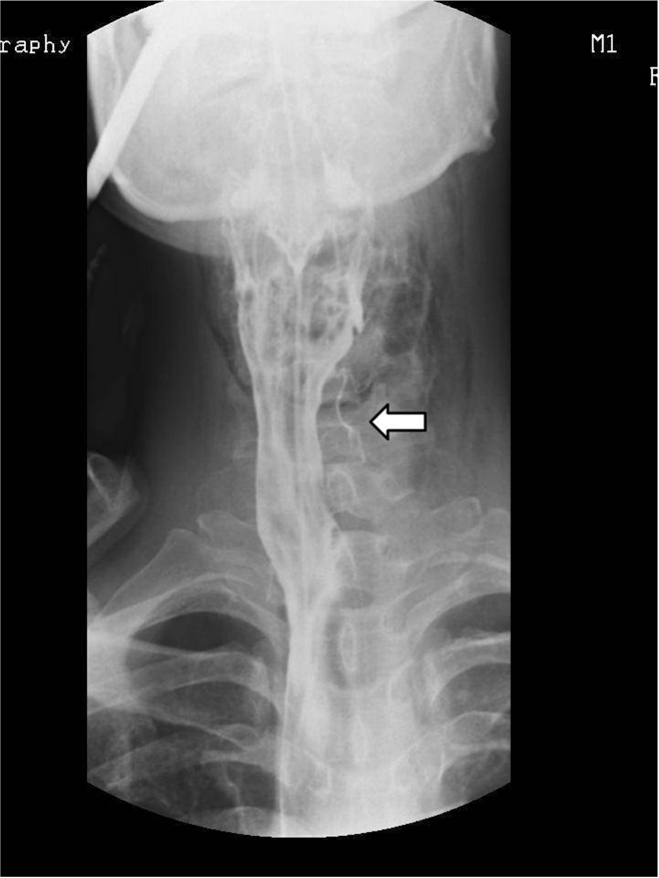
**(Arrow) A small leakage was observed on an esophagogram performed on the first postoperative day.**

**Figure 7:**
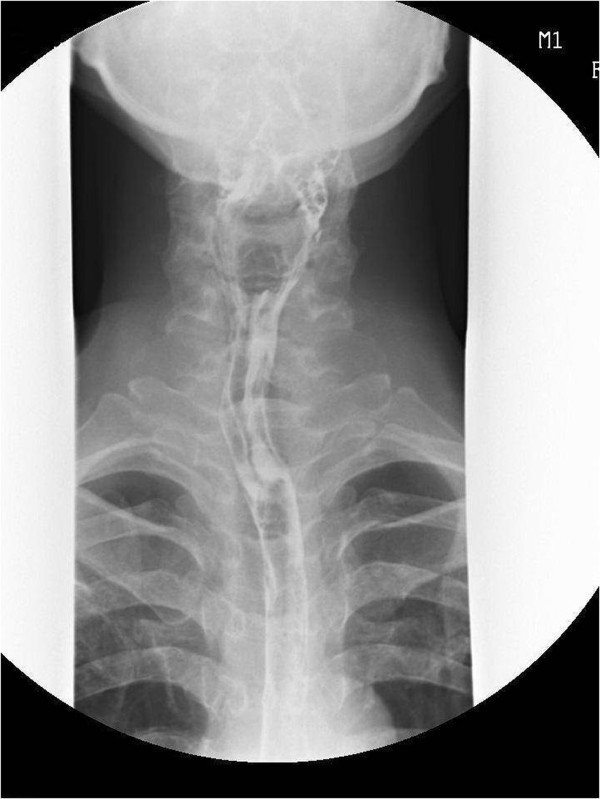
**Normal findings were observed on an esophagogram performed on the 10** ^**th**^ **postoperative day.**

To evaluate hypopharyngeal and esophageal damage, an esophagogram was obtained using a water-soluble contrast dye on the first postoperative day, and a small perforation in the hypopharyngeal region was identified. Accordingly, the patient received conservative therapy, including intravenous broad-range antibiotic administration and was placed on oral dietary restrictions. No specific findings or complications were detected via the esophagogram on the 10^th^ postoperative day, and the patient was subsequently discharged and is currently being followed up (Figures 6 and 7).

## Discussion

A toothbrush is a commonly used object in one’s daily life, but visiting the emergency room because of swallowing a toothbrush is rare; such a case may rarely occur in patients with mental retardation or a history of psychological disorders. Unlike a typical small foreign body, a toothbrush rarely passes through the gastrointestinal tract by itself; in most cases, the toothbrush becomes trapped in the esophagus or the stomach, but in some cases, it may even move down to the large intestine [1, 2]. Cases of injury to the oropharyngeal region caused by a broken toothbrush fragment in children have been reported [3]. Yet, even taking into account the psychiatric history of this patient, cases where a whole toothbrush is swallowed arbitrarily and ends up in the nasopharyngeal and hypopharyngeal regions are extremely rare.

Diagnosis of the foreign body is typically performed through a close examination of the patient’s medical history and a physical examination. However, when treating patients with mental disorders, consultation with hospital officials or guardians is also important. In cases of pointed or sharp foreign bodies, the foreign body may cause perforation of the esophagus and hypopharynx, and therefore additional imaging is necessary. In cases of small foreign bodies, and fish bones in particular, movement of the muscle associated with deglutition as well as inflammatory reactions of the adjacent tissues may move the foreign body, which may necessitate the use of neck CT or MRI rather than plain radiography [3–6].

Characteristics of a toothbrush shown on imaging include brush head plates where multiple lines of toothbrush hair are fixed in a parallel manner [7]. In this present case, a series of non-radiolucent contrast regions located inside the nasal cavity were visible upon Waters’ view and were confirmed on CT of the neck.

In this particular case, the foreign body was diagnosed through a medical history and physical examinations only, but imaging examinations were also performed for the possibility of associated damage to neighboring tissues. Moreover, an esophagogram using Gastrografin® was performed to identify hypopharyngeal damage.

A toothbrush part may become stuck in the esophagus or gastrointestinal tract upon swallowing, and serious complications such as a pressure ulcer, gastritis, mucosal damage, and perforation could follow [1]. Thus, immediate removal is required; if the toothbrush is found in the gastrointestinal tract without complications, endoscopic removal is possible [8].

In the present case, because of the firmly planted toothbrush that spanned the nasopharyngeal cavity and the hypopharynx and could not be moved, and also given the numerous major anatomical structures nearby, the removal was performed under general anesthesia. However, although the object was removed immediately, uvular laceration, hypopharynx perforation, and broad subcutaneous emphysema had already occurred upon swallowing the foreign body.

Despite the fact that antibiotic use may be controversial, with the exception of cases where the inflammatory response is severe, antibiotics were administered in this case to prevent complications given the suspicion of hypopharyngeal perforation [5, 9]. Furthermore, following a period of treatment including oral dietary restrictions and non-oral hydration, an esophagogram with Gastrografin® was used to verify that the damaged region around the perforation was safe.

It is believed that this case may be a rare example of its kind, in which a toothbrush spanning the nasopharynx and hypopharynx was removed via a surgical procedure and the accompanying hypopharynx perforation was treated using conservative treatment without additional problems.

## Conclusion

In patients with a history of psychological disorders, problems caused by unexpected foreign bodies may occur, as was the case with this patient, and a rapid diagnosis as well as treatment is imperative. The presence and state of a foreign body must be determined through a careful physical examination and imaging, followed by the immediate removal of the foreign body, all while keeping in mind the possibility of accompanying damage to nearby tissues.

## Consent

Written informed consent was obtained from the patient for publication of this case report and any accompanying images. A copy of the written consent is available for review by Editor-in-Chief of this journal.

## Abbreviations

CT: Computed tomography
MRI: Magnetic resonance imaging.
